# Values, decision-making and empirical bioethics: a conceptual model for empirically identifying and analyzing value judgements

**DOI:** 10.1007/s11017-023-09640-4

**Published:** 2023-08-17

**Authors:** Marcel Mertz, Ilvie Prince, Ines Pietschmann

**Affiliations:** 1 Institute for Ethics, History and Philosophy of Medicine, Hannover Medical School, Carl-Neuberg-Str. 1, 30625 Hannover, Germany; 2 Institute of Philosophy, Leibniz University Hannover, Hannover, Germany; 3 Department for Medical Ethics and History of Medicine, Goettingen University Medical Center, Goettingen, Germany

**Keywords:** Values, Value judgement, Decision-making, Empirical bioethics, Ethical theory, Animal research ethics

## Abstract

It can be assumed that value judgements, which are needed to judge what is ‘good’ or ‘better’ and what is ‘bad’ or ‘worse’, are involved in every decision-making process. The theoretical understanding and analysis of value judgements is, therefore, important in the context of bioethics, for example, to be able to ethically assess real decision-making processes in biomedical practice and make recommendations for improvements. However, real decision-making processes and the value judgements inherent in them must first be investigated empirically (‘empirical bioethics’). For this to succeed, what exactly a ‘value judgement’ is and of what components it might consist must initially be theoretically clarified. A corresponding conceptual model can then support or even enable empirical data collection and analysis and, above all, subsequent ethical analysis and evaluation. This paper, therefore, presents a value judgement model with its theoretical derivation. It also illustrates its application in an interview study of decision-making between animal experimentation and alternative methods in the context of biomedical research. Though the model itself can be theoretically deepened and extended, the application of the model works in general and helps to uncover what value judgements can enter into decision-making. However, the empirical methods, for example, qualitative interviews, can also be better oriented towards eliciting value judgements (as understood according to the model). Further applications of the model to other topics or by means of other empirical methods are conceivable.

## Introduction

### Value judgements and decision-making

It is fair to assume, theoretically as well as empirically, that virtually all decision-making in human affairs involves, in one way or another, value judgements (i.e., in a nutshell, judgements about what is ‘good’ or ‘bad’). Theoretically, decisions are, *inter alia*, intertwined with what the decision-maker deems important, valuable, or necessary [1, 2]. This all inevitably involves values; otherwise, choosing between several options would be quite impossible—why prefer option A to option B when there is no assessment possible about whether A is ‘better’ than B?

Empirically, the crucial role that values and value judgements can play in decision-making is not only true for one’s personal life [3, p. 9] or politics [4], but also for scientific research, even when sometimes not (sufficiently) acknowledged [5–8]. Values and value judgements are paramount [9] when, for example, setting a research focus, selecting methods, defining endpoints in clinical research, choosing a language and/or vocabulary, or considering possible (ethical, social) consequences (‘risks’) when accepting or rejecting a hypothesis (“inductive risk” [6]).

Moreover, value judgements are obviously necessary in medical or medical-related research. Central values, such as ‘health’ or ‘well-being,’ are always somehow part of decision-making processes—even if they are working in the ‘background’ of an actual decision situation. Health technology assessment is a perfect—and already intensively examined—example of this [10–13], as it incorporates manifold value judgements on quite different levels of assessment, appraisal, and decision-making. There are other decision-situations in medical and life sciences research where value judgements do not play a lesser role, but have not yet been as thoroughly regarded—for example, decisions on whether to conduct an animal experiment or to choose an alternative to it (we will return to this example later).

### Value judgements and empirical bioethics

However, even when one theoretically acknowledges the importance of value judgements in research contexts, the common problem in praxis is that, as Hofmann et al. put it:

[m]any of the value judgments are implicit or tacit, and, by not making them explicit, the illusion of scientific objectivity and neutrality is reinforced. However, by leaving these judgments implicit, they may cloak important value issues and controversies and, as such, frame or ‘bias’ the decision-making process. [10, p. 583].

A (biomedical) researcher thus obviously does not need to be (directly) aware of the reasoning structure of one of her/his value judgements. This poses a particular problem if one wants to *empirically* investigate decision-making processes in order to make an ethical assessment of these processes and the value judgements involved—as is often the case in so-called *empirical bioethics*.^1^

This is because theoretical presuppositions play a role in the empirical identification of value judgements via, for example, qualitative content analysis [14] of transcripts of an interview study. There, one must know what to ‘look for’ in a text in the first place, and has to have a theoretical approach for analyzing and interpreting text passages that entail value judgements. Presuppositions based on an understanding of the term ‘value judgment’ contribute to what is ‘seen’ and analyzed in texts; differences in the meanings of this term are not merely meta-ethically relevant. It thus matters whether value judgments are, for example, understood as the “preferences of persons or groups” that “complement data from observation” [15, p. 521] as an action-guiding instrument to steer behavior toward the best solution to the person’s problem which is empirically justified [16], or as kinds of *beliefs* that “are directed to objectivity” [17], p. 471]. Finally, one also needs to understand what exact part—or implication—of a text passage is evaluated ethically in the end.

In short, a clear *definition* and *conceptual model* of value judgments in the context of decision making are already needed to empirically identify such judgments (not just to analyze them ethically).

### Research questions and goals

However, in philosophy and ethics, literature concentrating on a more detailed analysis of the ‘nature’ of a value judgement is rather scarce,^2^ leading to a lack of sophisticated definitions and corresponding conceptual models. In the context of the planning of an interview study we conducted for an ELSI (ethical, legal, and social issues) project that focused on decision-making regarding alternative methods for animal experiments (“R2N-E1”),^3^ we therefore formulated two research questions for the theoretical part of this project:

1. What is a plausible definition of ‘value judgement’ that allows for the development of an empirically applicable model for identifying and analyzing value judgements in decision-making processes?
2. How can value judgements, according to this definition, be depicted theoretically in an empirically fruitful manner (conceptual model)?

These research questions have arisen in the context of a specific project in the field of animal research ethics that used interview techniques. However, the resulting ‘value judgement model’ can be relevant to all areas of bioethics where decisions and thus value judgments are investigated with socio-empirical studies. The model is therefore mainly a contribution to the *methods* of empirical bioethics and their practical operationalization. Nevertheless, it also contributes to the philosophical sharpening of the term ‘value judgment.’

In the following, firstly, the ‘value judgement model’ itself, along with the explicit definition of the term ‘value judgment’ will be presented. This will be followed by explanations of the model and the (philosophical) rationale for the definition. After that, the model will be applied to examples from the project about alternative methods for animal experiments. This article will end with a discussion of the strength and weaknesses the conceptual model and its application, with a word regarding its further use as part of the methods of empirical bioethics.

## Value judgement model

The conceptual model presented in the following is intended to reflect a (graphical) representation of the logical or even (hypothetical) causal relationships of components of a value judgment in a decision-making process. The model is based on our explicit definition of ‘value judgement:’

An explicit or (mostly) implicit evaluative conclusion (using ‘thin’ or ‘thick’ concepts) in relation to a general or concrete state of affairs (esp. action/decision) which is based on at least one evaluative and one descriptive premise and which is intended to, and can language-pragmatically fulfil, an evaluative function. (For explanations, see the “Values, judgements and value judgements—Elaborations” section below).

As a consequence, value judgements can be premises of a *practical syllogism* and, thus, part of the justification of an action/decision. Therefore, it is important that not only the actual components of a value judgment (e.g., evaluative premises) are represented in the model, but also some other elements or causal factors of decision-making in this context. So, for example, emotions, interests, or epistemic processes that sometimes can be difficult to delineate from a value judgment or one of its components should also be represented.

**Fig. 1:**
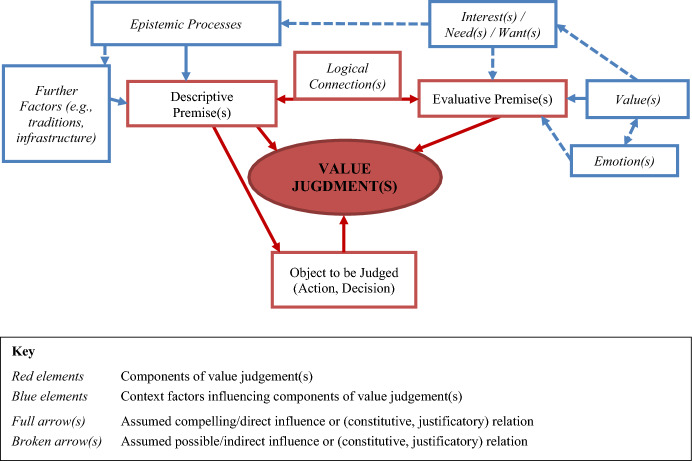
Value judgement model (adapted to value judgements in the context of animal experimentation research)

However, the model finally developed^4^ (see Fig. 1) does not claim to represent *all* factors or elements of a decision-making process. It is limited to those that have been considered particularly important, especially regarding the empirical application planned (see application examples below in the section “Example of an empirical application of the model: Value judgements in the choice between animal models and alternatives”). Furthermore, the model does not presuppose that one value judgement *alone* has to be sufficient to justify a particular decision/action. Different value judgements can play a role in concrete decision-making situations and may stand in opposition to each other. Thus, further arguments or complex argumentations, in which value judgements are only making up a part, might be necessary. Such argumentations might also contain trade-offs between different value judgements (i.e., involve weighing of (conflicting) value judgements). Basically, this implies that there will be another value judgement on a meta-level, referring to the value judgements about an actual action or decision situation; however, this goes beyond the model proposed here. For the model, it is only important to recognize which value judgements were (probably) involved in a decision-making process, not to determine how ‘powerful’ a particular judgement was for the final action or decision.

The elements of the model (see Fig. 1) are described in detail in the following:

**Value judgement(s), descriptive premise(s), evaluative premise(s), logical connection, object to be judged**

All the *red elements* in the figure are components of a value judgement as established when analyzing the concept ‘value judgement’ and developing the explicit definition (see “Values, judgements and value judgements—Elaborations” below). Descriptive and evaluative premises are regarded as central to every value judgement, and because of the justificatory/argumentative structure, some logical connection(s) between descriptive and evaluative premises is unavoidable. The ‘object to be judged’ by a value judgement can be an action or a decision; it is the object to which the value judgement is referring and to where its evaluative function aims. This object is necessarily represented in the descriptive premise(s).

**Value(s)**

Values (as characterized in the analysis) are crucial for building evaluative premises. They may also influence or constitute emotions and interests/needs, which, in turn, may also influence the building or acceptance of evaluative premises (see below).

**Emotion(s)**

The relationship between emotions and values is difficult: are values constitutive of (certain) emotions, or are (certain) emotions instead constitutive of values? This depends on how emotions are characterized. In addition to understanding emotions as mere affective phenomena, there are also views that regard emotions themselves as a kind of “judgement of value” [26]—even though emotions, when considered as judgements, are not understood in such a way that these judgements are always consciously or freely available (rather to the contrary), but can be articulated if necessary [27], p. 11]. However, a view that identifies emotions outright with *beliefs* is often rejected, because even if emotions may have some cognitive content, they are not *only judgements*, but characterized by their affective features [28]. This is an argument for not equating emotions with evaluative premises (which are, logically speaking, beliefs), and even less so for equating them directly with value judgements. Still, beliefs can be a cognitive precondition for emotions [27, p. 8]. Thus, a value (as a belief) can be such a precondition for the model. Nonetheless, in sum, the relationship between values and emotions remains debatable. Therefore, a possible mutual influence is depicted for the model.

**Interest(s)/need(s)/want(s)**

Like emotions, it is assumed that interests, needs, and wants influence evaluative premises or can be a further basis for them. *Interest* is the partaking of a person in another person, living being, thing, or an event; stemming from original, vital, or psychic drives or needs. They not only produce pleasure in something but also initiate actions and desires and are, therefore, generalized behavioral tendencies. There are interests that persist over time and, in turn, interests that change erratically. *Needs* are what a living being strives for to maintain, improve, and increase its life. *Wants* are often the motivational component which, together with beliefs and plans, play a central role in explaining actions and intentions [29]. They, in turn, can be influenced by values or even only be brought into being by values. For example, the desire for a certain form of fair treatment exists only—in the assumption of the model—against the background of a corresponding value of justice. Interests, for example, are usually accompanied by a positive valuing [30].

**Epistemic processes**

Epistemic processes have to do with how knowledge is gained and justified, especially in the context of scientific research and methodology. In this context, they are important for the generation (and further justification) of descriptive premises.

**Further factors**

This element of the model is deliberately broad and contains other factors necessary for the formation—or justification—of descriptive premises. In the context of research, these can include institutional or systemic preconditions of science, existing traditions of scientific working methods, or the infrastructure available (which, for example, enables animal experiments but not alternatives). In other applications of the value judgement model, of course, the factors may differ.

## Values, judgements, and value judgements—elaborations

However, is our explicit definition of a value judgment that forms the basis for our conceptual model convincing? If this definition falls, at least part of the model falls as well. The following elaborations are therefore intended to reflect the underlying analysis and justify the definition, and, *a fortiori*, the model proposed. The initial conceptual analysis of the basic terms ‘value’ and ‘judgement’—as separate components of the complex term ‘value judgement’ (following the standard procedure of de-compositional concept analysis (see [31, 32])—is also necessary to answer the first research question. Thereafter, the results of the analysis are combined, including inevitable theoretical decisions about how ‘value’ and ‘judgement’ *should* be understood when following this particular analysis. Thus, the aim of the analysis is *not* to provide a full-fledged theory of value or value judgements per se.

### Values

There are different characterizations of the meaning of ‘value’ in the context given. Value, in the broadest sense, is taken as something that goes hand in hand with the notion of “goodness” or “badness” [31]. It has to be considered that “…[t]here are many types of goodness, such as scientific, economic, technical, medical, professional, aesthetic, and moral goodness” [10, p. 580]. In other words, ‘value’ does not necessarily refer to a *moral* value.

Regardless of whether it is a moral value or not, a value is the “reason or the result of an evaluation, i.e., the preference of one action over another or, in general, of one object or fact over another” [34, p. 662; own translation]. Values act, thereby, as “conscious or unconscious orientation directives for human performance” and give “human existence meaning and direction” [35, pp. 528–529; own translation]. This all can be expressed in a concept-analytical definition provided by Burger [36, p. 69; based on 37], which is only slightly modified below:

For all* Z*,* Z* is a value iff (if-and-only-if):

- *Z* is an intentional object for an individual A (i.e., A may refer to it);
- *Z* refers to a state of affairs* X*;
- it is the case a) that X has certain qualities such that it is a good, and b) X counts for A as a good^5^ (whether X occurs or does not occur).

Condition (ii) of the analysis establishes an objective reference to a state of affairs, while conditions (i) and (iii) establish a subjective reference. However, it must be presupposed for condition (ii) that facts can be determined sufficiently intersubjectively or that the existence of a fact can be interpreted in a sufficiently consistent manner.^6^ An implication of condition (iiib) is that a value can, among others, act as an action-oriented directive, corresponding to the general characterization that was used as a starting point.

The ‘availability of clean water’ (in short, only ‘clean water’) can be used as a simple example of a value. ‘Clean water’ is an *intentional object* for an individual A, because she/he can consciously refer to it. ‘Clean water’ refers to a *state of affairs* in the empirical world, for example, whether the water available for individual A (e.g., from the tap or from a river) is not contaminated or similar. Since ‘clean water’ is important for a human being for health reasons, *inter alia*, it is a *good* for individual A, and individual A would count it as a good even if it was not available (i.e., the water was polluted). Therefore, ‘clean water’ is a *value*, which can be expressed as a desirable idea (‘It is desirable that there is clean water everywhere’), but also as an action-oriented directive (‘Clean water everywhere is a fact that should be worked towards’).

Values can be represented in a hierarchy, from more concrete values to more general, superordinate values, which, in most cases, also represent intrinsic values (‘values in themselves,’—i.e. something that is not only valuable for something else) and not extrinsic values (i.e. something that is valuable for something else). The example of ‘clean water’ is a relatively concrete and probably also extrinsic value.

From these considerations, the element “… *in relation to a general or concrete state of affairs (esp. action/decision) …*” from our above definition of ‘value judgment’ becomes understandable.

### Judgements

The term ‘judgement’ can be interpreted in philosophy in various ways: (i) psychological: ‘judgement’ as the result of a thought process; (ii) linguistically: ‘judgement’ as a (declarative) sentence; (iii) logical: ‘judgement’ as a conclusion; and (iv) ontologically: ‘judgement’ as a proposition or a fact (cf. [38]).

In the following, ‘judgement’ shall be used particularly in its logical meaning, i.e., as a result of a logical-argumentative context of interlinked statements. The *practical syllogism* can serve as a classic example of a judgement in the sense used.

According to the Aristotelian original, a practical syllogism consists of (i) a major premise containing a general principle, law, or maxim (a *prescriptive* or *evaluative* knowledge/belief component), (ii) a minor premise designating a certain concrete characteristic (*descriptive* knowledge/belief component), and (iii) a conclusion containing an action or possibly a request for action. In more modern accounts (see e.g., [37]), an additional (most often implicit) premise that describes the will or the wishes of a person (*volitive/conative* component) is also considered. For example:

- All humans should exercise.
- I am a human
- (I want to do what other humans should do).
- ∴ I exercise.

When applied to *moral justification*, the practical syllogism contains a general moral premise (i.e., also a prescriptive or evaluative premise), as a major premise, a concrete case in the minor premise, and the (moral) action or possible call to action (imperative) in the conclusion. For example:

- It is wrong to kill a human being.
- Paul is a human being.
- (I don’t want to do anything wrong).
- ∴ I will not kill Paul / Do not kill Paul!

The conclusion of such an argument is called a (moral) *action* judgement, which, however, can be used to clarify (logically) what a (moral) *value* judgement is.

As a result of these considerations, the element “… *which is based on at least one evaluative and one descriptive premise* …” in our proposed definition of 'value judgment' follows.

### Value judgements

Simply put, “[v]alue judgments are judgments about what is good” [10, p. 580]. However, given the several presuppositions discussed above, this is definitely *too* simply put, as a value judgement could also be a judgement about what is *bad* (not good). Value judgements are (conscious) *evaluations* and must be separated from mere *valuings*. Valuing refers here to the (immediate) liking, appreciation, or disliking of things, without much awareness that one (and why one) is valuing a specific thing (cf. [40]). The difference of value judgments from *preferences*, which are also commonly regarded as a kind of evaluation, is that the latter refer to the subjective and comparative (“X is better than Y”) (cf. [41]). Value judgements, on the other hand, are usually objective, or intersubjectively defendable,—or at least claim to be—and are not restricted to comparative evaluations.

‘Good’ and ‘bad’ in a value judgement can be given a more precise meaning in a given context, i.e., when it is said *how it is good or bad in a certain way*. Then, other (more) ‘thin’ concepts are legitimate *evaluative terms*, such as ‘right’ and ‘wrong,’ ‘just’ and ‘unjust,’ or ‘beautiful’ and ‘ugly.’ Additionally, the wide range of ‘thick’ (ethical) concepts, such as ‘tactful,’ ‘charitable,’ ‘cruel,’ ‘paternalistic,’ ‘altruistic,’ or even ‘valid’ when assessing the quality of an empirical study, may then be legitimate terms.^7^

When looking closer at the logical structure, a (moral) value judgement does not follow from a practical syllogism but is a judgement of a theoretical syllogism.^8^ Still, a (moral) value judgment can contain almost identical premises:

- It is wrong to kill a human being.
- Paul is a human being.
- ∴ It is wrong to kill Paul.

In comparison to the action judgement, the value judgement is missing the volitive or conative component.^9^ The major premise in the argumentative justification of a value judgement is an evaluative premise. Such a premise is distinguished linguistically and logically by using *evaluative propositional operators*. For example, generally ‘it is (morally) good that p’ or ‘it is (morally) bad that p.’

If a prescriptive premise is used (= reference to a norm), this refers implicitly to the evaluative premise on which it is based. Evaluative premises can refer directly to a (more concrete or more general) value (cf. [43, p. 12]), or indirectly via a norm. Therefore, an ‘evaluative premise’ may not automatically be equated with ‘value.’ Logically, a value can often be seen as a further premise, as in the case of examples above containing ‘life conservation,’ ‘enabling happiness/joy,’ or ‘existing as a self-determined individual’ (which is destroyed by killing). However, an evaluative premise can also be associated with certain values independently of the justification structure of a concrete value judgement (i.e., it does not necessarily have to be explicated as a separate premise).

These first considerations explain the element “*An explicit or (mostly) implicit evaluative conclusion (using ‘thin’ or ‘thick’ concepts)* …” in our definition of ‘value judgment’.

However, the justification of a value judgement also consists of descriptive premises – i.e., it would be wrong to call a value judgement *exclusively* ‘related to values’ or similar (see also [43, p. 13]). Neither should value judgements be equated with statements expressing emotional states ([43, p. 21]), although emotions may play a role in the formulation or acceptance of evaluative premises.

It is aggravating that descriptive statements can be ‘disguised’ value judgements; such sentences are often also referred to as being *crypto-normative* [22, 44]. Furthermore, value judgements can act in a similar way to descriptive statements. Although one can think about a boundary between description and evaluation *in abstracto*, in reality, many statements are—or at least seem to be—descriptive and evaluative simultaneously, probably also due to the ‘thick’ (ethical) concepts that are used.

Descriptive statements (premises) are substantiated by reference to everyday experience, scientific findings, or accepted law; evaluative statements (premises) are substantiated by reference to general valuation assumptions (= values, further evaluative premises). The boundary between the two types of statements is flexible and determined both by the beliefs about the scientific or unscientific character of certain opinions or theories and the scope of the given value system (which may or may not include, for example, rules for human behavior towards animals) (cf. [43, p. 173]).

Therefore, in the end, the *linguistic pragmatic function* for differentiation is sometimes decisive. Consider what the utterance of the judgement is supposed to achieve in the respective discursive context: should it just convey information (descriptive function) or should it (also) convey an evaluation (evaluative function)? To count as a value judgement, the conclusion of the argument must not only be able to fulfil an evaluative function. Rather, the evaluative function must be intended.

These second considerations make it clear why the definition of 'value judgment' contains the closing element of our above definition: “*… which is intended to, and can language-pragmatically fulfil, an evaluative function.*”

### Example of an empirical application of the model: value judgements in the choice between animal models and alternatives

In the following, we discuss how the model can be applied in empirical ethical research using a practical example based on data from our R2N-E1 project mentioned at the beginning (see also [45]), which provided the impetus for developing our value judgment model.

Value judgements inevitably play a role in deciding whether to use an animal model or an (possible) alternative. Various aspects of scientific validity (comparing established animal models and new alternative methods) are often weighed, on the one hand, and the possibility of reducing the suffering or distress of animals, on the other. However, the fact that such decisions are also based on value judgements, which are partly ethical, partly methodological (and partly even pragmatic), is not always made sufficiently evident in the biomedical sciences [45]. This can lead to decisions being seen as ‘without alternative;’ although, in the end, certain judgements—based on particular assumptions, values, and associated weighing processes—are behind them.

As an essential part of the R2N-E1 project, an interview study investigating the *structure* of value judgements in the decision-making about alternatives to animal experimentation was conducted. In the end, 13 bioscientists in basic and translational research who either (a) perform animal experiments themselves, (b) use alternative methods, or (c) at best both, were interviewed in 2020 in the form of a semi-structured interview. The interviews, which were conducted in German, were structured to invite narration of relevant decision-making situations (i.e., where the researchers themselves faced the decision to choose an animal model or an alternative, or where they at least observed such decision-making situations occurring with other researchers or research groups). The interviews were transcribed and processed for empirical and ethical analysis with methods of qualitative content analysis.^10^

The interviews provided rich narratives discussing, or at least mentioning, the reasons for choosing an animal or an alternative method. However, it became clear during our analysis of the interviews that not every element of the proposed value judgement model can be clearly extracted from the statements of the interview partners. In some cases, the value judgement itself was articulated, but not (all of) the premises; in others, the narrations only mentioned reasons, i.e., premises, but not the value judgement itself. Therefore, some of the elements according to the model often had to be *reconstructed*. This reconstruction was oriented mainly towards the idea of a ‘rational reconstruction.’ That is, it was about reconstructing the argumentation (in the sense of the value judgement model) in a rationally plausible and consistent way. Such a process always involves *interpretation*, and, depending on the specific narration of the interviewee, the interpretation was either relatively ‘close to the text’ or quite speculative (as there was virtually nothing in the narrative about any particular element of the model). The latter occurred mainly where it concerned the elements that have been called ‘Context factors influencing components of value judgement(s)’ (the ‘blue elements’ in the model, see Fig. 1), and those more related to the evaluative premise, i.e. ‘Interest(s)/Need(s)/Want(s),’ ‘Emotion(s),’ and ‘Value(s).’ The context factors that were more related to the descriptive premise (e.g., ‘Epistemic Process(es)’) were more likely to occur or were easier to reconstruct from the narratives.^11^

Personal values and interests were rarely communicated by the interviewees. Because of this, we have chosen to use a simplified tabular form for the actual evaluation of the interviews in which the value judgement is supported by its descriptive and evaluative premises (also see Table 1). The context factors were depicted in an additional category called *descriptive* and *evaluative backings*. *Backings* are, thus, mainly (further) justification of the premises, but also entail factors influencing the content of the premises (e.g., values or other factors, such as infrastructure or the work environment). Logically, these are other premises in an extended argumentation, but they are often implicitly thought about and, are rarely made more explicit than the value judgements themselves and their (immediate) descriptive and evaluative premises.

**Table 1:** Examples of value judgements analyzed on basis of the interview study

*Examples of value judgements*	*Examples of descriptive premises* (DP)	*‘The alternative method A has/does not have characteristics Z’*	*Examples of (possible)* *Descriptive Backings (DB)*	*Epistemic processes, infrastructure, work environment …*
*‘The alternative method A is good/better with regard to X (than the animal model/experiment B)’*	*Examples of Evaluative Premises (EP)*	*‘X is good/bad, right/wrong, etc.’*	*Examples of (possible)* *Evaluative Backings (EB)*	*Interest(s)/need(s), emotions, values …*
1. Alternative method A has less serious consequences (esp. for animals) when the experiments fail than animal experiment B (*)	DP: Alternative method A has the consequences* Z */ does not have the consequences* Z*’ (esp. for animals) when the experiments fail		DB: The animal experiments in this kind of research imply (mostly) problematic consequences (work environment)	
	EP: Serious consequences (esp. for animals) when experiments fail are bad (should be avoided)		EB: Animal welfare (value), efficiency (value)	
2. Alternative method A is not cruel (compared to the animal experiment B)	DP: Alternative method A is equivalent to the animal experiment in the field in terms of knowledge gain without inflicting* Z *on animals (e.g., inducing strokes in rats)		DB: It is necessary to do* Z *to animals (epistemic processes) to investigate this topic	
	EP: The animal experiments (in this kind of research) are cruel [= ethically bad] (*)		EB: Animal welfare (value)	
3. Animal experiment B is justifiable when no alternative method A is available and when it contributes to less harmful animal experiment conditions in the long run	DP: There was no alternative method A for the important research question that would have been worked on anyway		DB: Funding for cell culture research was discontinued	
	EP: One’s own handling of laboratory animals would be respectful (and, therefore, the best possible way to contribute to animal welfare). Respectful handling of laboratory animals should be transferred into the training of future researchers (*)		EB: Animal welfare (value), assigning an intrinsic value/moral status to an animal /avoiding the instrumentalization of animals (value)	

*Elements that were explicit in the interview

Based on the interviews, we present three examples of reconstructed value judgements, their premises, and their (probable, possible) backings in Table 1. An additional example will compare value judgements from several interviewees regarding the specific topic of (financial) *costs* of animal models vs. alternative methods (not depicted in Table 1). Elements that were explicit in the interviews are marked with an asterisk (*), though we have often paraphrased, ‘streamlined,’ and generalized the original wording of the propositions in order to make them more usable for an ethical analysis. Any direct quotes in the following text are from the interviews we conducted. They have only been translated into English here and partially shortened for their purpose as examples.

In the examples depicted in Table 1, descriptive premises were based mostly on a characterization of the research question to be investigated and the suitability of animal models and their alternatives to answer this question. In addition, there was a description of different consequences depending on the experiment and the use of the animal. This can be demonstrated in formulations, as in the *first example*: Alternative method A has the consequences* Z */ has not the consequences* Z *(esp. for animals) when the experiments fail. The evaluative premise is often connected to the content of the descriptive premise and evaluates, for example, the outcomes described in ethical terms.

The interviewee from the *second example* supported her or his descriptive premise with statements such as “we used to do an animal experiment for this kind of research” but “we can replace the animal experiment equivalently in terms of knowledge gain.” The evaluative premise is based on the scientists’ judgement that “the animal experiments (in this kind of research) are cruel.” In contrast to the rather clear premises, the backings are more speculative and constructed such that values like animal welfare, avoidance of cruelty, truth, common good, professional success informs, etc. justify value judgements and their premises, respectively.

The *third example* is interesting because the value judgement itself was not as clear to identify as in the former examples but had to be reconstructed from a variety of evaluative and descriptive premises. The descriptive premise is based on the scientist’s claim that “there was no option to do a cell culture experiment for the specific research question at hand.” It was added that “someone would do the experiment anyway.” The scientist made a distinction in the evaluative premise between his or her own respectful behavior towards animals and the potentially disrespectful behavior of other scientists. The chance to share this respectful experience of animal experimentation was also seen as a chance to educate students about the morally responsible handling of animals in laboratories. These elements build our reconstructed value judgement. Backings, in this case, are the available funding options that influenced the research possibilities and the scientist’s wishes to improve the handling of animals.

The model can also be useful when analyzing different value judgments that relate to the same issue, such as *cost*, which will be discussed as an additional *fourth example*. One interviewee said that alternatives such as cell culture experiments are just cheaper, which is an obvious reason to choose them. This can be paraphrased as value judgment: ‘Alternative method A is cheaper (compared to the animal experiment B).’ An evident evaluative premise might be ‘A method that is cheaper is favorable (to other methods that are more expensive).’ This evaluative premise is likely to be shared even by those who *disagree* with the value judgment in a specific case (in contrast to the evaluative premise in the third example above that a particular experiment is ‘cruel’).

In fact, another interviewee did not, at first sight, agree with the value judgment, essentially saying: ‘Alternative method A is not necessarily cheaper (compared to the animal experiment B).’ The difference is not explained by the evaluative premise, but mainly at the level of the descriptive premises; the interviewee talked about so-called *organoids,*^12^ which probably rather represent a ‘high-end’ and therefore more costly alternative method.

However, the first mentioned interviewee made clear that the choice nevertheless depends quite clearly on whether the research question can be answered at all with the alternative; otherwise, irrespective to the costs, the animal model must be chosen. The descriptive premise would probably reflect that. For example, ‘Alternative A allows investigating the same research question as animal experiment B.’^13^

At the level of backings, statements about the cost of a specific alternative are thus likely to be related to the type of alternative method, funding structures, and (existing) infrastructure (e.g., costs due to housing of the animals). Statements might also have to do with the interests and values of the researchers, whether they consider something to be ‘cheap’ or ‘expensive’ (the interviews in general indicated that someone who has a strong interest in alternative methods, even if only scientifically, may value an corresponding investment differently than a researcher who does not have this interest). This became clear in an interview in which the interviewee referred to her/his own values or professional values as a scientist (evaluative backings). In her/his opinion, it is not the goal of the scientist to avoid or replace animal experiments (but rather to do ‘good science’). Therefore, alternative methods must offer an *advantage* over animal experiments in order to be chosen. This could include, as an example, (financial) costs.

From the example, one can see that both differences in value judgments on the topic of ‘cost’ and differences in the justification of a comparable value judgment will depend less on evaluative premises than on descriptive premises. Nevertheless, the evaluative premise(s), interests, values, and the professional self-image of researchers can be significant as backings; and must, therefore, also be taken into account in order to be able to classify and evaluate the value judgment with regard to costs.

The conceptual model highlights the fact that the value judgment is only the tip of the iceberg and that the various components of the value judgment must also be taken into account for an ethical evaluation.

## Strength and weaknesses of the value judgement model

### Strength and weaknesses as a theoretical approach

The model inevitably has certain theoretical limitations. Individual components, for example, of a value judgement, such as ‘interests,’ ‘emotions,’ and especially ‘epistemic processes,’ are not (yet) sufficiently tied back to more extensive philosophical or, where plausible, psychological theories. Furthermore, the model cannot completely *describe* or *explain* real decisions or processes of decision-making. This means that some aspects always remain excluded, which could perhaps also play a role in actual decision-making in reality.

These limitations are pragmatic and exist in order for the model to remain applicable as an *instrument*, especially as an analytical and/or interpretative tool within the framework of empirical-ethical research.^14^ Thus, the model does not want to claim (and cannot claim) to be a full-fledged philosophical theory of value judgements, nor to be an empirically adequate theory of decision-making. The model can only (and wants only to) claim to be able to look particularly at the aspect of *value judgements* from an *argumentative* point of view. This, however, is precisely a point of view that is particularly significant in an *ethical* approach—in contrast to an approach, for example, from axiology or psychology. Therefore, the strength of the model must be seen less in its—extendable—theoretical pervasiveness and more in how well it is ultimately able to support the analysis of ethical decision-making and evaluation. In this regard, it must be acknowledged that our value judgment model is not the only way to make implicit value judgments (more) explicit. However, on the one hand, it is a plausible way to do this, and on the other hand, it also allows us to uncover the argumentative structure and to put the individual components and factors of a decision-making process into a systematic nexus. Moreover, the model also contributes to a further general theoretical understanding of value judgements—notwithstanding its applicability as an analytical or interpretative framework for empirical interview data.

### Strength and weaknesses as an empirical-ethical approach

The concrete object of knowledge (e.g., reason, argument, preference, opinion) in empirical-ethical projects is not always sufficiently identified nor theoretically determined in more detail. Our model presented here was one of the rather few attempts to provide such a theoretical basis even before the empirical data collection and analysis was carried out.

To be clearer about what the object of knowledge is (how it is understood) is not only a theoretical-reflexive concern. It also determines which empirical methods are used and how exactly, for example, interviews should be conducted and how the interview questions should be designed. The latter, of course, should be done in a way that evokes relevant statements from the interview partners about the object of knowledge. However, this presupposes that one has a sufficiently clear understanding of the object of knowledge (in this case, of value judgements and their components). In this way, interview questions can be targeted from the outset so that their answers are more likely to contain something relevant about value judgements or their components. Additionally, a model like our value judgement model presented here is also significant when it comes to classifying, evaluating, and—argumentatively—interpreting the interview data (via statements, narratives). While the value of the former (formulation of questions) cannot be discussed further here, the value of the latter (analysis, interpretation) can, at least, be exemplified (see Table 1 and related paragraphs).

The actual application of the model within the R2N-E1 interview study also showed various limitations. Among other things, it revealed the need to adapt the interview technique better in order to gain narratives/statements that are more focused on certain components of the value judgement model. Thus, the interviews actually conducted often contained too few clear statements to fill out all components of the value judgement model, which is why some components then had to be supplemented in a rational and reconstructive way in our analysis.

However, even improving the formulation of interview questions and techniques will have limits, since one’s own values are not always evident, first requiring abstraction and reflection. This probably has little place in the practice of the interviewees. Such reflections may, however, be triggered by qualitative interviews.^15^

Despite all the limitations, the application of the model as an analytical tool can still make clear what is ‘always already presupposed’ in descriptive and evaluative assumptions in real-life decision-making; here we used the example of deciding between animal models and alternative methods. The application of our model, thus, definitely allows a certain insight into the weighing and arguing of life scientists in their social reality.

This, in turn, is relevant for an *ethical* evaluation of decision-making: some of the reasons (premises or backings) identified with the model in the R2N-E1 example are ethical (i.e., can be traced back to ethical norms/principles), while others are not. The latter does not mean that they are automatically *un*-ethical. They may simply refer to extrinsic values that indirectly promote intrinsic values. But they may invite particularly critical scrutiny that might be important for improving research practice(s) regarding animal experimentation and alternatives in basic and translational biomedical research. However, ‘ethical reasons’ are not necessarily convincing. They are, after all, usually the subject of an *ethical debate*.

Overall, the value of our model is, above all, to make the reasons and their argumentative connections transparent against an ethical background; thereby allowing a (better) critical examination of the respective decision-making process.

## Outlook and future applications

The value judgement model presented is a first attempt not only to describe value judgements and some of their components merely theoretically, but, above all, to be able to identify, analyze, and interpret them more precisely in empirical-ethical studies. As a first attempt, it is fraught with ‘teething troubles.’ Still, its successful application to the R2N-E1 project demonstrates its inherent potential to improve empirical-ethical research theoretically and methodologically. Our model does this by orienting both the empirical data collection (which object of knowledge is to be investigated exactly?) and the (subsequent) empirical analysis (to which ‘headings’ does, for example, an identified reason belong: to ‘emotions,’ ‘epistemic processes,’ etc.?) more clearly. Last but not least, the model and its application supports (or even enables) the normative or evaluative analysis and interpretative work (e.g., the identification of descriptive and evaluative premises and the ethical evaluation of these premises).

In the future, further theoretical refinements and philosophical or psychological deepening of individual components would be conceivable. Depending on the purpose, the focus could be on individual components of the model or additional components to be incorporated. The application of the model to other topics than presented here (animal models vs. alternative models) is, of course, possible (and encouraged!). Furthermore, the model could also be applied in the context of other empirical methods, such as document analyzes instead of interview studies.

Regardless of which direction may be taken in the further use of the value judgement model, it has become clear that the possibilities and limits of a theoretical model—as an analysis and interpretation tool—such as the one presented, can only be determined in actual practical use, i.e., in the context of empirical-ethical research, not exclusively through philosophical reflection.

## Footnotes

1 *Empirical bioethics* is proposed to be understood “[…] as an interdisciplinary activity in which empirical social scientific analysis is integrated with ethical analysis in order to draw normative conclusions” [18, p. 1]. Not all researchers engaging in empirical research in bioethics will agree with this narrow understanding of empirical bioethics, as it may constrict the aims of empirical research projects in bioethics (which can be rather broad, see e.g. [19–21]). Nonetheless, it is assumed in the following that empirical research in bioethics “is not an end in itself,” but rather “a required step towards a normative conclusion or statement” [22, p. 1]. Since only few of the empirical data that would be relevant from an ethical point of view are generated by other disciplines (sociologists or psychologists have often other, discipline-driven interests), it is necessary for bioethics itself to collect and analyze this data.

2 This is not true for literature on, for example, ‘value’ or on the discussion of whether value judgements can be understood in an objectivist manner [17, 23], whether value is a mere expression of emotions [24] or whether an expression of attitudes is a necessary or sufficient condition for a value judgement [25].

3 The R2N-E1 project was part of the R2N consortium (“R2N – ‘Replace’ and ‘Reduce’ from Niedersachsen (Lower Saxony),” https://r2n.eu/). The interview study has not yet been published.

4 The model was developed by using the aforementioned definition of a value judgement as a starting point, which, in turn, refers back to specific theoretical characterizations of ‘value,’ ‘judgement,’ and ‘value judgement.’ Additionally, further theoretical considerations and past experience when analyzing value-based decisions have been incorporated. Altogether, there was a critical exchange between four people on the plausibility and possible applicability of the model. Finally, several fictional test applications of the model were conducted to check for factual applicability.

5 This can also be read meta-ethically in such a way that values result from the (empirical) appreciation, thus, the interests of individuals.

6 It is debatable whether this condition already must imply a cognitivist approach to meta-ethics. Non-cognitivist approaches could also agree with condition (ii) and still uphold non-cognitivism by referring to the subjectivity of condition (iii). Regarding especially the empirical analysis of actual value judgements, however, it should not make a pragmatic difference; thus, one can remain agnostic concerning cognitivism/non-cognitivism at this point.

7 The difference between ‘thin’ and ‘thick’ (ethical) concepts goes back to Bernard Williams [42] and is, as is often the case in philosophy, not uncontroversial. While ‘thin’ concepts are considered to be only evaluative, ‘thick’ concepts are often considered to be evaluative *and* descriptive, at least richer on details than ‘thin’ concepts. For the analysis of value judgements presented here, the following is assumed: i) Whether the conclusion of the argument (= value judgement) contains a ‘thin’ or ‘thick’ concept is related to the content of the premises (i.e. the more detailed they are, the ‘thicker’ the concepts in a value judgement may be, which is close to a ‘separabilist view’ regarding ‘thick’ concepts); ii) Thus the difference between ‘thin’ and ‘thick’ concepts is one of degree, not of kind; iii) Broadening up the range of evaluative terms beyond the usual ‘thin’ concepts is also particularly fruitful for an empirical analysis of value judgements.

8 It is difficult to reconcile the process of drawing conclusions for value judgements with the model of practical syllogism. One would have to assume that the value judgement is *directly* related to an action or its omission.

9 Nonetheless, a value judgement still provides *motives* for action, i.e., it gives a (partial) answer to why one has performed an action or made a decision (cf. [43, p. 9]).

10 We are working on a further publication presenting, among other things, the results of the interview study. Details on the aims, method, and the results of the interview study can then be found there.

11 This may say something about i) the interviewees (that, following the stereotype/cliché of a natural scientist, evaluative elements tend not to be told, as researchers in these fields are often less aware of them), ii) the interview method used, iii) the value judgement model and the limits of its application, or about all of the above.

12 An organoid is a simplified ‘mini-organ’ grown in vitro, but with basic functions of the respective organ, e.g., liver or lung.

13 A premise that, in specific cases, is actually quite debatable. The interviewee gave very detailed examples where she/he sees hardly any alternatives to animal experimentation.

14 It may be helpful to consider at this point that, overall, a *pragmatism*-oriented view (see e.g. [46]) of theories is taken here. In other words, theories would basically not (or not so much) be understood as approximately accurate ‘representations’ of reality, but as instruments (‘tools’) to structure and interpret experience and enable insights for ameliorating future actions.

15 Some interviewees of the pre-interviews, which were conducted for the development and testing of the interview guide for the actual qualitative interview study, had communicated afterwards that they had not thought about such decision-making situations and their reasons beforehand (i.e., that they had only done so during the interview, after being confronted with the respective questions).
